# Optimizing Injection Molding Parameters of Different Halloysites Type-Reinforced Thermoplastic Polyurethane Nanocomposites via Taguchi Complemented with ANOVA

**DOI:** 10.3390/ma9110947

**Published:** 2016-11-22

**Authors:** Tayser Sumer Gaaz, Abu Bakar Sulong, Abdul Amir H. Kadhum, Mohamed H. Nassir, Ahmed A. Al-Amiery

**Affiliations:** 1 Department of Mechanical & Materials Engineering, Faculty of Engineering & Built Environment, University Kebangsaan Malaysia, Bangi 43600, Selangor, Malaysia; 2 Department of Machinery Equipment Engineering Techniques, Technical College Al-Musaib, Al-Furat Al-Awsat Technical University, Al-Musaib 51009, Babil, Iraq; 3 Department of Chemical & Process Engineering, Faculty of Engineering & Built Environment, Universiti Kebangsaan Malaysia, Bangi 43600, Selangor, Malaysia; amir@eng.ukm.my (A.A.H.K.); dr.ahmed1975@gmail.com (A.A.A.-A.); 4 Program of Chemical Engineering, Taylor’s University-Lakeside Campus, Subang Jaya 47500, Selangor, Malaysia; mohamedh.nassir@taylors.edu.my

**Keywords:** nanocomposite, injection parameter, mechanical, physical properties

## Abstract

Halloysite nanotubes-thermoplastic polyurethane (HNTs-TPU) nanocomposites are attractive products due to increasing demands for specialized materials. This study attempts to optimize the parameters for injection just before marketing. The study shows the importance of the preparation of the samples and how well these parameters play their roles in the injection. The control parameters for injection are carefully determined to examine the mechanical properties and the density of the HNTs-TPU nanocomposites. Three types of modified HNTs were used as untreated HNTs (*u*HNTs), sulfuric acid treated (*a*HNTs) and a combined treatment of polyvinyl alcohol (PVA)-sodium dodecyl sulfate (SDS)-malonic acid (MA) (treatment (*m*HNTs)). It was found that *m*HNTs have the most influential effect of producing HNTs-TPU nanocomposites with the best qualities. One possible reason for this extraordinary result is the effect of SDS as a disperser and MA as a crosslinker between HNTs and PVA. For the highest tensile strength, the control parameters are demonstrated at 150 °C (injection temperature), 8 bar (injection pressure), 30 °C (mold temperature), 8 min (injection time), 2 wt % (HNTs loading) and *m*HNT (HNTs type). Meanwhile, the optimized combination of the levels for all six control parameters that provide the highest Young’s modulus and highest density was found to be 150 °C (injection temperature), 8 bar (injection pressure), 32 °C (mold temperature), 8 min (injection time), 3 wt % (HNTs loading) and *m*HNT (HNTs type). For the best tensile strain, the six control parameters are found to be 160 °C (injection temperature), 8 bar (injection pressure), 32 °C (mold temperature), 8 min (injection time), 2 wt % (HNTs loading) and *m*HNT (HNTs type). For the highest hardness, the best parameters are 140 °C (injection temperature), 6 bar (injection pressure), 30 °C (mold temperature), 8 min (injection time), 2 wt % (HNTs loading) and *m*HNT (HNTs type). The analyses are carried out by coordinating Taguchi and ANOVA approaches. Seemingly, *m*HNTs has shown its very important role in the resulting product.

## 1. Introduction

During the last three decades, an important shift has been carried out from traditional alloys and minerals to plastics. The shift was primarily due to easy processing, less cost and the compatibility of plastic material compared to the traditional ones. The importance is also extended to include production of biocompatible materials [1]. Plastic materials are currently used in piping, packaging, automobiles and, most importantly, in the medical field. Recently, plastics are found to be very competitive materials in the field of injection molding where the last process for industrial manufacturing is taking place. Injection molding provides the ultimate route for dealing with a polymer or blends of copolymers. Briefly, engineering polymer includes vast and broad arrays of types of polymers, additives, properties and production processing conditions. Injection molding is a process through which materials are injected into a mold with or without a host material. The injection process depends on a variety of variables, such as filling time, packing pressure, packing time, cooling time, mold temperature, injection pressure and cooling rates.

Injection is an old technology; however, it has undergone great development in the last 60 years. Injection molding is commonly a very stable process; however, it could undergo some drawbacks, such as discontinuity and internal interaction of several variables, which might demolish the product’s good qualities. Injection molding requires appropriate parameters, which, in turn, could dramatically change the physical and mechanical properties of the product. Maximizing the injection molding parameters is another important step that can be performed via several techniques, such as the design of experiment (DOE) by Taguchi. The optimizing process is used to set the injection molding parameters in order to reduce the number of experiments and to ensure the best quality [2].

Injection is a very complex process [3] due to the difficulty of controlling many factors, which include the type of the plastics, additives, temperature, pressure and the cooling/heating rate. Consequently, the designing engineers found a powerful procedure to overcome most of these difficulties by using computer-aided engineer (CAE) software. By using CAE, a trial injection is made followed by a continually reiterating design throughout the simulation process. As the need for a variety of plastics with specific properties as a common practice, the software has undergone a series of improvements [4]. Setting injection parameters depends partially on previous knowledge about the polymer and deterministically on the concept of trial and error. However, in this regard, rheology could add very important basic and fundamental knowledge about the parameters that strongly influence the injection process of plastic parts. Molten polymers are generally non-Newtonian fluids whose characterizations are very crucial in the final setting of the parameters. It is very important to note that polymer additives have a very strong influence on the rheological behavior of the molten polymer [5]. Additives are used to enhance the physical and mechanical properties of the produced polymers, such as resistance to scratches and altering the surface visual characteristics [6]. The tensile, thermal and wettability properties, of the nanocomposites were studied as a function of halloysite nanotubes (HNTs) content and found that it is strongly dependent on both the nature of the polymer and the HNT functionalization [6]. The process of additives to the polymers is growing very quickly, and most of the production relies on certain experimental procedures, rather than on the simulation process [7]. Previous research on injection molding has covered aspects that range from the effect of the parameters, the influence of additives, the cost and others. Rahman et al. [8] suggested that the hollow frame for windows was better for a lower cost than the solid frame. The injection could be performed at high pressure; however, this factor is necessary, but it could be considered as a drawback. Injection molding and the subsequent parameters and their appropriate levels are commonly used to control the properties of the injected materials, such as thermoplastic polyurethane (TPU) and nanotubes. TPU is a unique polymeric material with special physio-chemical properties, and its versatility provides the possibility for various applications [8]. Finnigan et al. [9] was the first report in the literature of the preparation of layered silicate-TPU nanocomposites. Since then, a significant number of papers has been published regarding the processing, characterization and fabrication of TPU nanocomposites for high performance and multifunctions [10]. However, most of these nanocomposites have nanoclays [11,12,13,14] and carbon nanotubes (CNTs) as fillers [15,16,17].

The optimization process is an essential process in industry and research in order to characterize the best use of the parameters and to avoid an unnecessarily excessive number of experiments. In this regard, Dr. Genichi Taguchi introduced a technique to address these two points for better outcomes of what are commonly known as responses [2,18,19]. The Taguchi method is a combination of mathematical and statistical techniques, where both the control parameters and their relevant responses are mixed to exercise the optimization process [20]. Orthogonal arrays and signal-to-noise (S/N or SNR) ratios are the major tools used in the Taguchi method and emphasize the consideration of quality in product and process design [21]. Because of these considerations, application of the Taguchi method, the S/N and the analysis of variance (ANOVA) seem to be a more practical approaches to the statistical DOE than other methods, which appear to be more complicated [22]. The Taguchi method was developed by systematically allocating factors and levels to suitable orthogonal arrays, then performing an analysis of the S/N and ANOVA to determine the optimal combination of parameters, to validate the results and identify the significant parameters that affect the quality [23]. This article presents a detailed method for such a calculation that can be used as a reference for researchers/engineers to build an approach using available software, such as Microsoft Office Excel [2]. In the Taguchi designs, the robustness of the control factors is used to identify the reduction of variability by minimizing the effects of the uncontrolled factors, which are known as the noise factors. The noise is a natural result from all errors encountering the experimental procedure, whether they are originated from mishandling measurements or due to unavoidable electronics devices. The higher value of S/N means the minimum effect of the noise factor. Taguchi’s approach includes two steps of optimization where the S/N signal is used to identify the control factors and secondly to move the mean to target a smaller or no effect of the S/N ratio. Choosing the level of the S/N ratio to a certain level depends on the goal of the experiment. In Minitab, there are four levels of S/N ratios, as explained in Table 1.

The selection of the control parameters and their applicable levels (minimum two) depends on the physical properties of the mixture components [24]. The responses are chosen based on the goal of the research under consideration. In this work, the responses are chosen to address the mechanical and the physical properties. The mechanical properties include the tensile strength, Young’s modulus, tensile strain and the hardness (Scale Shore A), while the density is the only physical property investigated. All mechanical properties are well known, and they were discussed in great numbers of research [25]. However, the hardness was seldom investigated or discussed. The hardness of a material is the resistance of its surface to penetration. The indentation caused by a standard size and shape at the surface of the elastomer is known as hardness. By comparing a small initial force and much larger force, the hardness can be measured. The Shore A scale for measuring hardness is very common global wise [26]. The aim of this work is to optimize the injection molding parameters for halloysite nanotubes-thermoplastic polyurethane (HNTs-TPU) nanocomposites through the Taguchi method. The use of HNT in this study as a nano-filler could be attributed to the HNTs’ tabular microstructure nature, high thermal resistance, unique crystallization behavior and the credibility of improving the mechanical properties of TPU based on previous studies [3]. The Taguchi method phase is the most important design one, which serves the objective of determining the optimal injection molding parameters to achieve the optimized parameters for the tensile, compression, hardness and density. The relationship between the control factors (injection temperature, injection pressure, mold temperature, injection time, HNTs loading and HNTs type) and output response factors (tensile, hardness, and density test) is thoroughly identified.

## 2. Materials and Methods

### 2.1. Materials

TPU was purchased from Global Innovations-polycarbonates Bayer material science AG, D-51368 Leverkusen. The physical properties of TPU include a tensile strength of 20 MPa, a density of 1224 kg/m^3^ and a melting temperature of 190 °C. HNTs were obtained from Natural Nano, Inc., New York, NY, USA, in powder form of an average size of 20 nm, a surface area of 65 m^2^/g, a pore volume of 1.25 m^3^/g, density of 2510 kg/m^3^, a refractive index of 1.54 and chemically is composed of O in SiO_2_ (61.19%), Al in Al_2_O_3_ (18.11%), Si in SiO_2_ (20.11%) and some impurities of about 0.50%. The sodium dodecyl sulfate (SDS), the disperser of molecular weight 288.38, was obtained from BioShop Canada Inc. (Burlington, ON, Canada); polyvinyl alcohol (PVA), the additive of molecular weight between 89,000 and 98,000, was purchased from Sigma-Aldrich, Saint Louis, MO, USA, and malonic acid (as a cross linker of CH_3_H_4_O_4_) was ordered from Sigma-Aldrich, Saint Louis, MO, USA.

### 2.2. Instrumentation

FESEM, Model ZEISS SUPRA 55-VP (Manufacturer, Konigsallee, Germany) with a magnification 10,000×, was used to investigate and view small structures on the surface of HNTs-TPU nanocomposites. The mixture of the HNTs-TPU nanocomposites was performed with a Brabender mixer (Model W 50 EHT) Corder PL 2000 compounder equipped with a 50-cm^3^ kneader chamber. For the preparation of specimens for testing, the injection apparatus DSM Xplore molding injection machine was used. The temperature of the heating chamber of 10 cm^3^ can be raised up to 350 °C. To investigate tensile strength and strain, an Instron Universal Testing Machine (INSTRON 5567) was used. The hardness of elastomer samples was measured using a Durometer provided with an “A” scale for soft materials and a “D” scale for materials of higher hardness. The test procedure was in line with D2240 [27]. The density was determined using the apparent loss by immersion test D792 [28].

### 2.3. Preparation of the Samples

**Figure 1:**
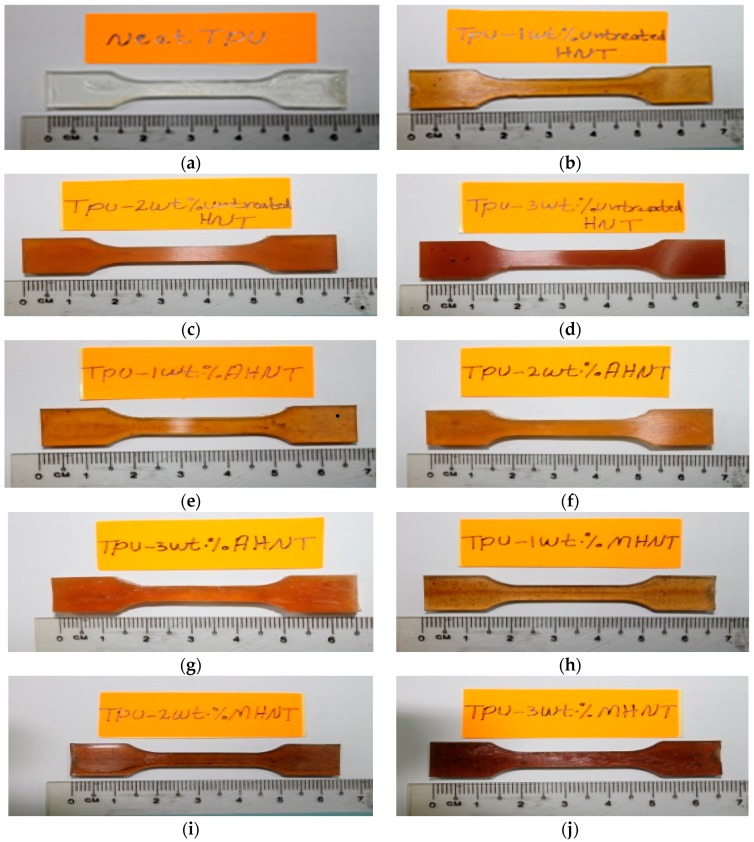
Samples of (**a**) thermoplastic polyurethane (TPU) matrix, (**b**–**d**) 1, 2, 3 wt % untreated halloysite nanotubes (*u*HNTs)-TPU nanocomposites, (**e**–**g**) 1, 2, 3 wt % acid treated HNTs (*a*HNTs)-TPU nanocomposites, (**h**–**j**) 1, 2, 3 wt % modified HNTs (*m*HNTs)-TPU nanocomposites.

The TPU spheres were dried in an oven at 80 °C to dehydrate water. Figure 1a shows the standard first sample after TPU is directly injected into the mold. The other samples, totaling nine, are prepared according to three different procedures. As a preliminary condition, HNTs and TPU were dried separately in an oven at a temperature of 80 °C for 12 h [29] to get rid of possible absorbed water due to storage. The first patch of three samples was prepared by mixing 0.5 g, 1.0 g and 1.5 g of HNTs and 49.5 g, 49.0 g and 48.5 g TPU to form 1, 2, 3 wt % HNTs-TPU nanocomposites, respectively. The three samples are labelled with 1, 2 and 3 wt % *u*HNT-TPU, where *u* refers to untreated HNTs, as shown in Figure 1b–d. The second patch was prepared by dissolving 15 g HNTs in 100 mL 3M sulfuric acid, and the mixture was kept at 90 °C and mixed at a rate of 200 rpm for 8 h. The sulfuric acid-treated HNTs were added to TPU at the same percentage mentioned for the first patch. Three samples shown in Figure 1e–g were prepared and labelled with 1, 2, 3 wt % *a*HNTs-TPU, where sulfuric acid treated (*a*) refers to the HNTs treatment. The third patch was prepared by creating a mixture of 1 g HNTs and 50 g distilled water and adding 0.10 g SDS (dispersion), 0.10 g PVA and 0.10 g of malonic acid (MA) (crosslinker). Again, three samples shown in Figure 1h–j were prepared following the same procedure mentioned for the first and the second patch. The three samples were labelled with 1, 2, 3 wt % *m*HNTs-TPU nanocomposites, where *m* refers to the modified HNTs-PVA crosslinked MA.

### 2.4. Taguchi Experiment

The injection was carried out in the injection mold at control parameters of temperature (140, 150 and 160 °C), pressure (4, 6 and 8 bar), mold temperature (28, 30 and 32 °C), injection time (4, 6 and 8 min), HNTs loading (1, 2 and 3 wt %) and HNTs type (*u*HNTs, *a*HNTs, *m*HNTs). It seems that all parameters are well defined except the injection time. The injection time is the time required by the machine from inserting the sample in the chamber until injection. The above levels of the process parameters were selected according to the data available in the literature [30,31] and the data recommended by the manufacturers. The selected injection molding process parameters along with their levels are given in Table 2. The optimized parameters using the DOE by Taguchi are tabulated in Table 3. The interactions between the parameters were not considered in this study [30,31,32]. The use of the experimental layout (L_27_ (3^13^)) model was carried out by four steps, involving the selection of the number of parameters, their appropriate levels and, finally, the experimental layout (L_27_ (3^13^)), which is suggested by the Minitab software program (16, Bizit Systems, Woodlands, Singapore). 

**Figure 2:**
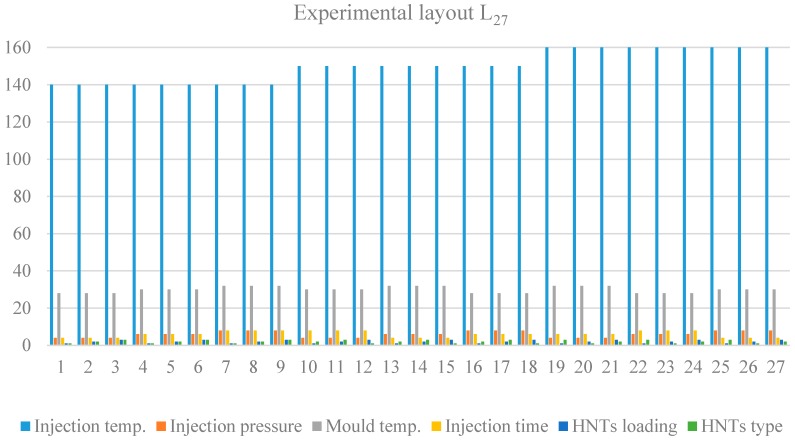
The experimental layout (L_27_ (3^13^)) Taguchi model for experimentation.

The experimental layout of the optimization is shown in Appendix A, while the results are shown graphically in Figure 2, which provides all parameters that could be used for theexperimental work.

## 3. Results and Discussion

The signal-to-noise (S/N) ratio is used to compare the level of the desired signal to the level of background noise. If S/N is greater than 1.0, the intensity of the signal is greater than the noise. In ANOVA, S/N is defined as the reciprocal of the coefficient of variation or simply the ratio of the mean to the standard deviation. The determination of S/N was performed according to the criterion of “larger-is-better” explained in Table 1. S/N was determined for the tensile strength, Young’s modulus, tensile strain, hardness Shore A and density. The ultimate stage of Taguchi method is to verify the predicted results via confirmation on the optimum set of parameters. In addition, more analyses are needed to determine the significance of each parameter and its contribution to each response. Such an analysis could be performed by employing the ANOVA approach.

### 3.1. Experimental Results

**Figure 3:**
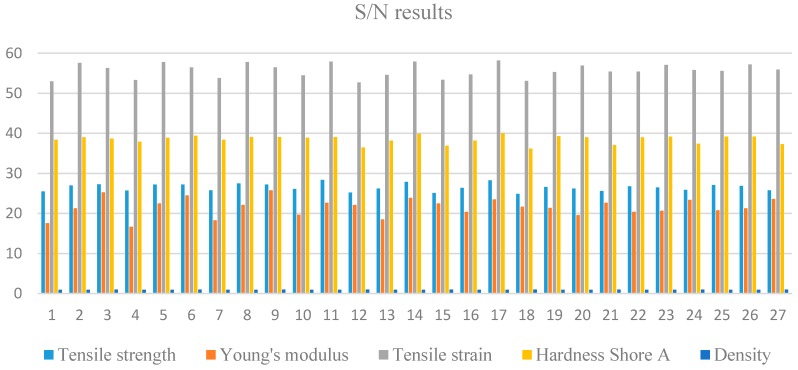
S/N results for the five responses.

Samples preparation has already been explained earlier. The experimental determination of tensile strength, Young’s modulus, tensile strain, hardness Shore A and density for thermoplastic polyurethane (TPU) matrix, *u*HNTs-TPU nanocomposites, *a*HNTs-TPU nanocomposites and *m*HNTs-TPU nanocomposites was repeated three times as required by DOE (Taguchi method) for all of the suggested parameters, and then, the average of each was determined and tabulated in Appendix B and depicted in Figure 3. In addition to the average values of each of the 27 experiments listed in Appendix B and depicted here by Figure 3, S/N ratios were determined using the relevant equation for larger is the better choice as shown in Table 1. The experimental results for all five responses are tabulated in Appendix B. The results were treated by software where S/N ratios were calculated and averaged. Appendix B contains, side-to-side, the actual average results of the TPU matrix.

### 3.2. Analysis Based on TPU Matrix Results

The following analysis relies on the real values of the responses without considering S/N values because there is no such figure for the TPU matrix. The highest, $x_{h}$, and lowest, $x_{l}$, of the experimental average results taken from Appendix B are tabulated in Table 3. The average results of the TPU matrix, $x_{o},$ are considered as the standard for calculating the maximum percentage variation according to, $x_{max}=\left(x_{h}-x_{o}\right)/x_{o}\%,$ and the minimum percentage variation, $x_{min}=\left(x_{l}-x_{o}\right)/x_{o}\%$. The absolute variation, $\left|x_{abs}\right|,$ between the highest, $x_{max}$, and the lowest variation, $x_{min}$, is calculated as follows: $\left|x_{abs}\right|=\left|x_{max}\right|-\left|x_{min}\right|$. The tensile strength is obviously the most important parameter. The absolute variation between the highest and the lowest measured values is calculated and found to be 48.6%. The information in Appendix B reveals that the injection temperature is applied for the same sample, which suggests that the injection temperature has no influence on the absolute variation of 48.6% between the highest and the lowest. All other control parameters have shown different applications. The data show that more pressure (8 bars instead of 4 bars) and more HNTs loading (3 instead of 2 wt %) cause a reduction in the tensile strength. The other parameters have shown the opposite influence as the reduction of molding temperature was reduced from 30 down to 28 °C and the reduction in injection time from 8 min down to 6 min. Young’s modulus, which is related to the tensile strength up to the yield point, shows different reactions towards the control parameters with the exception of injection time and HNTs type, where both remain unchanged compared to the tensile strength response. To obtain higher Young’s modulus, the molding temperature has to increase from 30 to 32 °C, and HNTs loading has to increase from 2 to 3 wt % and *m*HNTs. For the purposes of a good sample with good tensile strain, the injection temperature at 150 °C seems to have no influence on the highest or lowest measured values. However, higher pressure (8 bar instead of 4 bar) is better for *m*HNTs. The results show that lower injection time (6 min instead of 8 min) and lower HNTs loading (2 wt % instead of 3 wt %) are suitable for perfect nanocomposites, which can be used for packaging and other similar applications. The HNTs type continues to influence all mechanical and physical properties. In the case of a thin layer of the nanocomposites with high hardness, as measured by Shore A standards, the absolute variation between the highest and lowest hardness is 67.1%. The injection temperature, injection pressure, mold temperature at 150 °C, 8 bar, 28 °C and 6 min remain unchanged, respectively. The HNTs loading of 2 wt % is better than 3 wt %, and *m*HNTs still prevails over the other two HNTs types.

### 3.3. Analysis Based on S/N Results

Table 4 contains the highest and lowest averaged values based on the data in Appendix B for the five responses predicted by larger-is-better S/N estimation. All experimental measurements are subject to an error caused by direct observations or electronic measuring devices. When electronic devices are involved in the measurement, the error is referred to the noise developed by these devices emerging from the nature of the electronic components and possible amplification or filtration. The measurement could rely on the change in the environment around the experiment settings, such as temperature change, humidity and other unavoidable factors. Consequently, the exact value of any response contains an error that can be described by the S/N ratio. S/N ratio is defined as the ratio of the power of the signal, $P_{signal},$ whose amplitude is $A_{signal}$ to the power of the background noise, $P_{noise},$ whose amplitude is $A_{noise}$, as in Equation (1):

(1) $$\frac{S}{N}=\frac{P_{signal}}{P_{noise}}={\left(\frac{A_{signal}}{A_{noise}}\right)}^{2}$$

The signal and its relevant noise are measured within the same conditions in order to express these measurements in terms of their variances (Var.) or standard deviations (σ) as follows:

(2) $$\frac{S}{N}=\frac{\sigma_{signal}^{2}}{\sigma_{noise}^{2}}$$

The highest and lowest averaged values of the five responses considered in this work are tabulated in Table 4 with their relative percentage change along with the applied control parameters and their relevant levels. The relative percentage changes of the results could be used as indicators to show the effect of the modifications imposed on HNTs prior to being mixed with TPU. The parameters under investigation (responses) of tensile strength, Young’s modulus, tensile strain, hardness and the density have shown positive changes of 12.2%, 35.2%, 22%, 29.9% and nearly 1%, respectively. Focusing on the control parameters shows that the highest averaged values of the responses are influenced by the *m*HNT type.

Other levels of the control parameters have different contributions for producing the highest averaged values. An injection temperature of 150 °C (Level II) appeared three times compared to 140 °C (Level I), which appeared twice. The injection pressure of 8 bar (Level III) appeared four times compared to 4 bar (Level I), which appeared only once. The three levels of the molding temperature imposed in the injection process appeared as follows: 28 °C (twice), 30 °C (once) and 32 °C (twice), respectively. Only two out of the three levels of injection time appeared for the highest values: twice for 6 min (Level II) and three times for 8 min (Level III). HNTs loading with TPU appeared twice for 2 wt % loading (Level II) and twice for 3 wt % loading (Level III). A brief analysis of the S/N ratios’ outcome clearly shows that the influence of the level of each control parameter is different, except the *m*HNTs (five times), followed equally by injection temperature and injection pressure (four times), then injection time and HNTs loading (three times each).

### 3.4. Analysis Based on ANOVA

ANOVA is one of the most important statistical tools to analyze the differences among the means of the group based on their variances. In this analysis, the variance, not the mean of a particular variable, is grouped into components, which are related to different sources of variations. The *t*-test is the statistical test where the means of three or more groups are tested to see whether they are equal or not. The *t*-test compares these means for statistical significance set normally at 5% (95% or less is accepted).

#### 3.4.1. Effect of the Levels of the Control Parameters

**Figure 4:**
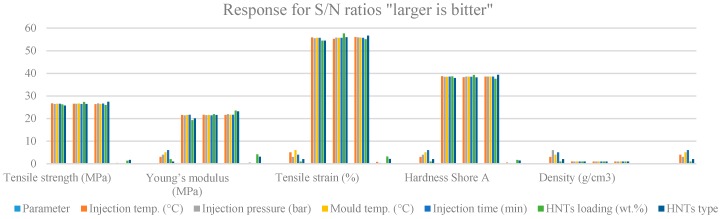
Response for S/N ratios “larger is better”.

Based on Appendix C and its relevant Figure 4, the effects of each level of the control parameters on the measurement of each response are tabulated based on estimated S/N ratios. Each control parameter has three levels as depicted in Table 2. The responses include the tensile strength, Young’s modulus, tensile strain, hardness (Shore A) and the density. The control parameters include injection temperature, injection pressure, mold temperature, HNTs loading (1, 2 and 3 wt %) and HNTs type (*u*HNTs, *a*HNTs and *m*HNTs). Only the highest obtained measurements are taken into account based on S/N estimation. Table 5 also contains Delta, Δ, which measures the arithmetic difference between the highest and the lowest averaged values, and the rank, which measures the influence of each control parameter in each level on each response. The highest delta represents the smallest response, which indicates that the result is better as the treatment causes better outcome compared to the lowest measured value. For the highest delta Δ (smallest rank), HNTs loading is the most influential parameter for all control parameters, except for the tensile strength, where the HNTs loading was replaced by HNTs type. On the contrary, the least influence of the control parameters for the responses appeared with injection time (tensile strength, tensile strain and HNTs type) followed by mold temperature (Young’s modulus) and injection pressure (hardness). The result of the hardness, where the least influential parameter is the injection pressure and density, suggests conflict with the traditional thinking that the pressure has a direct influence on the density and that injection time is not related. However, the measurement and subsequent analysis show a different outcome. Examining the results of the hardness and the density in Appendix C reveals that HNTs loading has the highest influence, not the pressure as expected. Seemingly, HNTs loading through which the vacancies in TPU are more important gives rise for higher density.

#### 3.4.2. Effect of the F-Ratio and Contribution

ANOVA, set at a *p*-value of 5%, of the tensile strength, Young’s modulus, tensile strain, hardness (shore A) and density for the TPU matrix, *u*HNTs, *a*HNTs and *m*HNTs are shown in Table 5. ANOVA analysis includes the sum of square (SS), degree of freedom (DF), mean square (MS), F-ratio and the percentage contribution of each factor. The only parameters that are critical for interpretation are the F-ratio and the contribution. The F-ratio is calculated by dividing MS by the mean square of error (MSE), and it is always positive. If the F-ratio is large, then the *p*-value is small, which means that the results are statistically significant. The second important factor is the percentage contribution of each of the control parameters, which means the highest is the most effective parameter. In this work, there are five responses, which are tested under the influence of six control parameters as explained earlier. Apparently, the responses are influenced primarily by the 1, 2 and 3 wt % HNTs-TPU nanocomposites, as shown in Table 6. The least significant effect of the control parameters is shown at the molding temperature and injection time. The results have not been signified by other researchers, whose focus was on the individual effect rather than the comprehensive effect.

#### 2.4.3. Graphical Analysis by S/N Means

**Figure 5:**
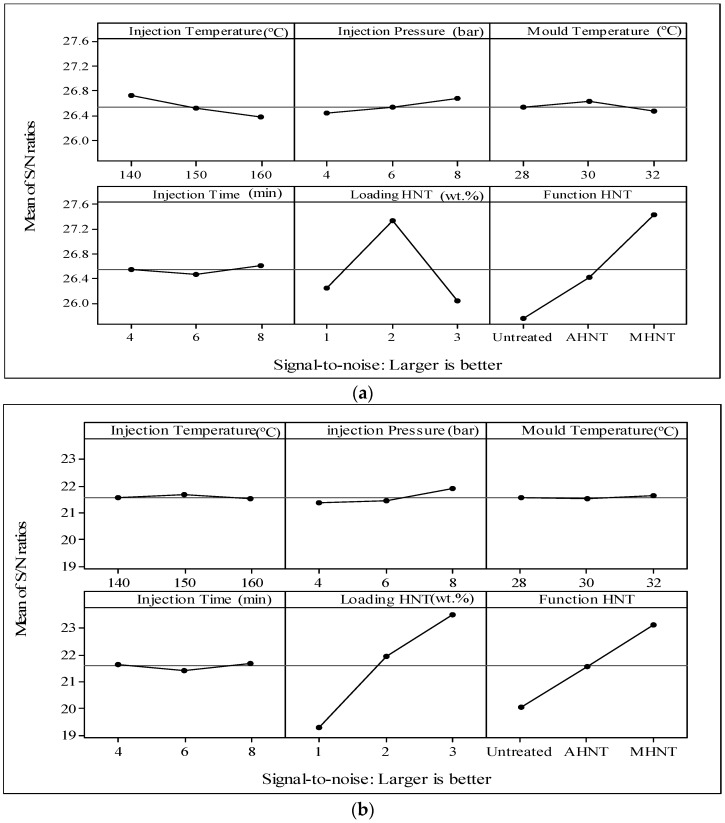

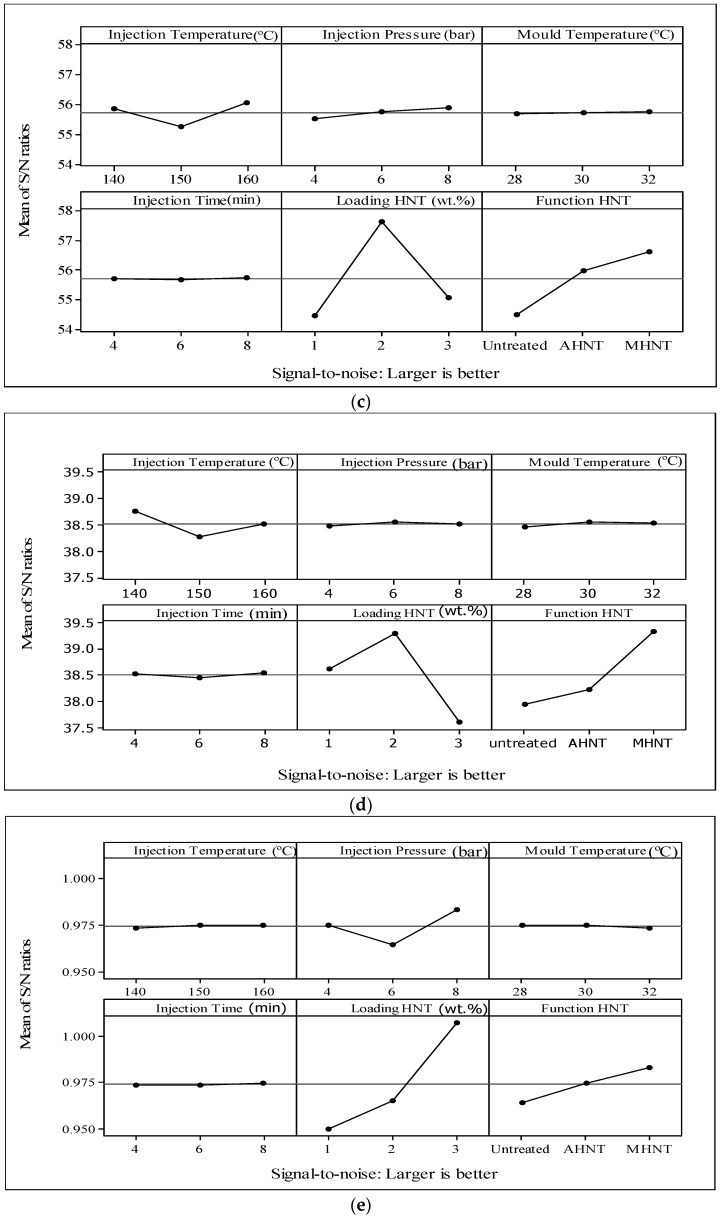
The effect of the levels of the control parameters on the responses: (**a**) tensile strength; (**b**) Young’s modulus; (**c**) tensile strain; (**d**) hardness; and (**e**) density.

Another approach for analyzing ANOVA results was considered by plotting the mean of S/N ratio of each of the five responses versus the three levels of I, II and III of the control parameters tabulated in Appendix C. S/N values are calculated under the criteria of “larger is better”, as explained in Table 1. The detailed results of all control parameters are shown in Figure 5. The results clearly show that the effect of Level I of the control parameters has the lowest contribution on the tensile strength and hardness (injection temperature of 140 °C). Level II of the control parameters has relatively more effects on the responses. The injection temperature of 150 °C and mold temperature of 30 °C influence the tensile strength; the injection temperature of 150 °C influences the Young’s modulus; loading at 2 wt % HNTs-TPU nanocomposites influences the tensile strain; the injection pressure of 6 bar, the mold temperature of 30 °C and HNTs loading at 2 wt % influence the hardness; and injection temperature of 150 °C and a mold temperature of 30 °C influence the density. The analysis by ANOVA is consistent despite the fact that each type of analysis serves a certain purpose. Collectively, ANOVA has proven its reliability, consistency and simplicity. The most important part of ANOVA lies in the contribution of the level on the response result. By acknowledging the contribution, one can easily set the parameters chronically and prepare samples accordingly expecting the best product.

### 3.5. Fractured Surfaces Characterization of Selected Samples

**Figure 6:**
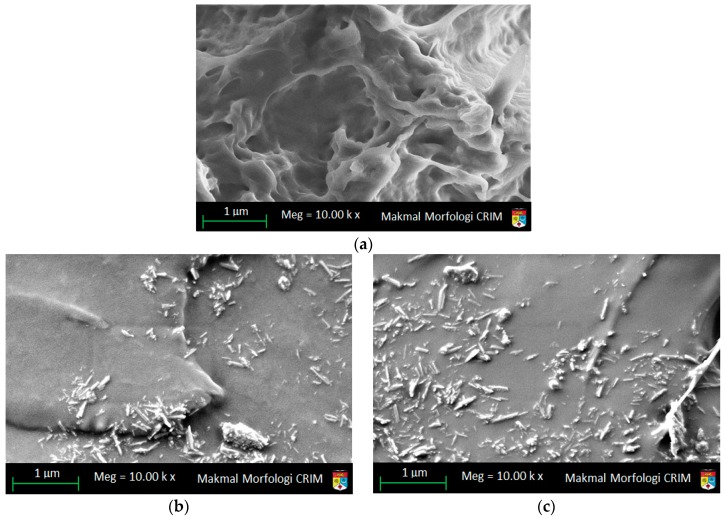

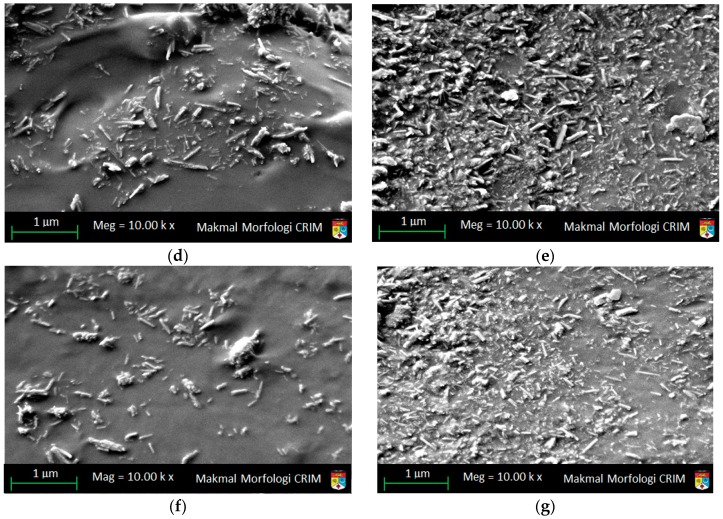
FESEM (10,000×) of (**a**) TPU matrix; (**b**) 1 wt % *u*HNTs-TPU nanocomposites; (**c**) 3 wt % *u*HNTs-TPU nanocomposites; (**d**) 1 wt % *a*HNTs-TPU nanocomposites; (**e**) 3 wt % *a*HNTs-TPU nanocomposites; (**f**) 1 wt % *m*HNTs-TPU nanocomposites; and (**g**) 3 wt % *m*HNT-TPU nanocomposites.

Seven samples were selected including the TPU matrix for FESEM investigation, as shown in Figure 6. The fractured surface is obtained at the break-off condition of the sample under axial stress. The fractured surface of the TPU matrix is shown in Figure 6a. The surface does not show irregularities, which seemingly suggests that TPU after injection is well-maintained regarding the existence of bubbles. For 1 wt % *u*HNTs-TPU nanocomposites, the fractured surfaces show the presence of *u*HNTs distributed in the matrix, as depicted in Figure 6b,c. The amount of *u*HNTs type on the fractured surface of 3 wt % *u*HNTs type is nearly three times that on the surface of 1 wt % *u*HNTs, as shown in Figure 6b,c, respectively. An in-depth investigation of Figure 6b,c reveals that cavities are significantly reduced in both size and number. For the 6 wt % *u*HNTs-TPU nanocomposites, the surface looks smoother than of that of 1 wt % *u*HNTs-TPU nanocomposites. The cavities shown on the fractured surface of the TPU matrix disappeared or their sizes were significantly reduced. The disappearing of cavities suggests that the tensile strength becomes better than that of the TPU matrix. This result agrees with the findings of the tensile strength and the hardness tabulated in Appendix B as the tensile strength increased from 18.2 to 26.3 MPa and the hardness increased from 64.4 to 100.4 due to the modification. FESEM images of the 1% and 3% *a*HNTs-TPU nanocomposites are shown in Figure 6d,e. The irregularities of the *a*HNTs’ fractured surface are significantly reduced as the distribution of HNTs becomes very clear. When *a*HNTs reaches 3 wt %, the surface becomes distinctive because of HNTs’ exfoliation due to sulfuric acid [33]. FESEM images of 1 and 3 wt % *m*HNTs-TPU nanocomposites are shown in Figure 6f,g. The existence of PVA is not clear through FESEM; however, its effect is expected due to the good dispersion of HNTs inside the polymer [34]. For 1 wt % *m*HNTs-TPU nanocomposites, the dispersion of HNTs has become clear as the surface at fracture shows almost no cavities, suggesting that almost no cavities as depicted in Figure 6f. For 3 wt % *m*HNTs-TPU nanocomposites, the distribution of *m*HNTs is very clear, and the *m*HNTs are largely broken.

## 4. Conclusions

Injection molding has been used to finalize products for marketing. Prior to injection, samples have to be prepared according to the optimization process, which is carried out independently. Another stage of optimization is performed prior to the injection process. The molding machine is equipped with sophisticated software; however, selecting parameters for optimization is not a part of the software. In this study, six control parameters (injection temperature, injection pressure, mold temperature, injection time, HNTs loading and HNTs type) were chosen to optimize five responses (tensile strength, Young’s modulus, tensile strain, hardness and density). The optimization does not depend only on the control parameters, but also depends on the level of each parameter. It was found that the control parameters and their suitable levels could be utilized as a guide for determining the qualities and purposes needed from the nanocomposite. The dispersion of HNTs is a very influential and effective approach in enhancing all responses while the levels play another important role for the best production. The other control parameters at certain levels have their own positive influence, which was measured by a combination of analyses, including Taguchi and ANOVA. Next to HNTs loading and HNTs type, it was found that injection the pressure of 8 bar is the most influential parameter, as it appeared four times followed equally well by the injection temperature of Level II (150 °C) and the injection time of Level III (8 min). The results are supported by FESEM, which shows clearly the dispersion of HNTs. All of these features have a direct impact on the quality of the nanocomposites regarding the mechanical and physical properties.

## Figures and Tables

**Table 1:** S/N ratio and experimental goals.

S/N Ratio	Experimental Goal	Data Characteristics	S/N Formula
Larger is better	Maximizing response	Positive	$-10\log\left(\sum^{\text{​}}\frac{1}{y^{2}}\right)/n$
Nominal is best	Targeting noise in standard deviation σ	Positive, zero, or negative	$-10\log\sigma^{2}$
Nominal is best (default)	Targeting noise in the mean (y) and standard deviation σ	Non-negative or possibly zero	$10\log\left(\frac{{\bar{y}}^{2}}{\sigma^{2}}\right)$
Smaller is best	Minimizing response	Non-negative, targeting zero	$-10\log\frac{\sum y^{2}}{n}$

**Table 2:** The parameters for three levels of the selected factors [30,31].

Factors	Level 1	Level 2	Level 3
Injection temperature (°C)	140	150	160
Injection pressure (bar)	4	6	8
Mold temperature (°C)	28	30	32
Injection time (min)	4	6	8
HNTs loading (wt %)	1	2	3
HNTs type	*u*HNTs	*a*HNTs	*m*HNTs

**Table 3:** Absolute variation of the actual results.

	Tensile Strength $x_{o}=17.7$ MPa		Young’s Modulus $x_{o}=2.3$ MPa		Tensile Strain $x_{o}=430$%		Hardness Shore A $x_{o}=55.45$		Density $x_{o}=1.112$ g/cm^3^	
Highest $x_{h}$	26.3		19.4		819.6		100.4		1.125	
Lowest $x_{l}$	17.8		6.8		431.1		64.4		1.113	
Absolute $\left|x_{abs}\right|$	48.6%		547%		90.3%		67.1%		≈0	
	max	min	max	min	max	min	max	min	max	min
Run	11	18	9	4	17	12	17	18	9	4
Injection temp.	150	150	140	140	150	150	150	150	140	140
Inj. Pressure	4	8	8	6	8	4	8	8	8	6
Mold temp.	30	28	32	30	28	30	28	28	32	30
Injection time	8	6	8	6	6	8	6	6	8	6
HNTs loading	2	3	3	1	2	3	2	3	3	1
HNTs type	*m*HNTs	*u*HNTs	*m*HNTs	*u*HNTs	*m*HNTs	*u*HNTs	*m*HNTs	*u*HNTs	*m*HNTs	*u*HNTs

**Table 4:** The variation of the responses.

Variation	Tensile Strength		Young’s Modulus		Tensile Strain		Hardness Shore A		Density	
Maximum	28.4		25.8		53.9		100.4		1.125	
Minimum	24.9		16.7		52.7		70.4		1.113	
Change %	12.2		35.2		2.2		29.9		≈ 1	
Variation	max	min	max	min	max	min	max	min	max	min
Run	11	18	9	4	17	12	17	18	9	4
Injection temperature	150	150	140	140	150	150	150	150	140	140
Injection pressure	4	8	8	6	8	4	8	8	8	6
Mold temperature	30	28	32	30	28	30	28	28	32	30
Injection time	8	6	8	6	6	8	6	6	8	6
HNTs loading	2	3	3	1	2	3	2	3	3	1
HNTs type	*m*HNTs	*u*HNTs	*m*HNTs	*u*HNTs	*m*HNTs	*u*HNTs	*m*HNTs	*u*HNTs	*m*HNTs	*u*HNTs

**Table 5:** ANOVA average on mechanical and physical properties.

Response	Parameter	Injection Temp. °C	Injection Pressure Bar	Mold Temp. °C	Injection Time (min)	HNTs (wt %)	HNTs Type	Error %
Tensile Strength (MPa)	SS	11.10	4.60	2.23	1.55	163.30	235.47	22.25
	DF	2	2	2	2	2	2	68
	MS = SS/DF	5.55	2.30	1.11	0.77	81.65	117.74	0.32
	F Ratio	16.94	7.041	3.39	2.36	249.48	359.72	1
	% Contribution	2.37	0.896	0.34	0.20	36.92	53.31	5.94
Young’s Modulus (MPa)	SS	6.35	8.77	0.82	2.52	452.27	256.91	48.46
	DF	2	2	2	2	2	2	68
	MS = SS/DF	3.17	4.39	0.41	1.26	226.13	128.45	0.71
	F Ratio	4.45	6.15	0.58	1.77	317.29	180.23	1
	% Contribution	0.636	0.95	−0.08	0.14	58.09	32.92	7.34
Tensile Strain (%)	SS	28,552.92	8232.141	108.29	135.34	815,792.9	281,665.9	70,253.45
	DF	2	2	2	2	2	2	68
	MS = SS/DF	14,276.46	4116.071	54.15	67.67	407,896.4	140,832.9	1033.13
	F Ratio	13.82	3.98	0.05	0.075	394.81	136.32	1
	% Contribution	2.19	0.512	-0.16	-0.16	67.54	23.21	6.86
Hardness Shore A	SS	197.87	4.54	12.24	6.83	3320.07	2647.80	2647.79
	DF	2	2	2	2	2	2	68
	MS = SS/DF	98.934	2.271	6.12	3.41	1660.04	1323.89	14.92
	F Ratio	6.63	0.15	0.41	0.23	111.25	88.72	1
	% Contribution	2.33	−0.35	−0.24	−0.32	45.67	36.34	16.57
Density (g/nm^3^)	SS	8.89 × 10^−7^	8.08 × 10^−5^	8.88 × 10^−7^	2.22 × 10^−7^	0.001	8.09 × 10^−5^	0
	DF	2	2	2	2	2	2	68
	MS = SS/DF	4.44 × 10^−7^	4.04 × 10^−5^	4.44 × 10^−7^	1.11 × 10^−7^	4.0 × 10^−4^	4.04 × 10^−5^	2.16
	F Ratio	0.201	18.643	0.20	0.05	184.693	18.64	1
	% Contribution	−0.31	6.88	−0.31	−0.37	71.63	6.88	15.59

**Table 6:** The highest and lowest effect of control parameters on responses.

Parameter	Tensile Strength		Young Modulus		Tensile Strain		Hardness		Density	
Highest and lowest	Highest	Lowest	Highest	Lowest	Highest	Lowest	Highest	Lowest	Highest	Lowest
Injection temperature (°C)										√
Injection pressure (bar)								√		
Mold temperature (°C)		√		√		√				
Injection time (min)		√		√		√		√		√
HNTs loading (%)	√		√		√		√		√	
HNTs type	√		√		√		√		√	

## Appendix A

**Table A1:** Experimental layout as per the L_27_ (3^13^) model.

Experimental No.	Injection Temp. (°C)	Injection Pressure (bar)	Mold Temp. (°C)	Injection Time (min)	HNTs Loading (wt %)	HNTs Type
1	140	4	28	4	1	*u*HNTs
2	140	4	28	4	2	*a*HNTs
3	140	4	28	4	3	*m*HNTs
4	140	6	30	6	1	*u*HNTs
5	140	6	30	6	2	*a*HNTs
6	140	6	30	6	3	*m*HNTs
7	140	8	32	8	1	*u*HNTs
8	140	8	32	8	2	*a*HNTs
9	140	8	32	8	3	*m*HNTs
10	150	4	30	8	1	*a*HNTs
11	150	4	30	8	2	*m*HNTs
12	150	4	30	8	3	*u*HNTs
13	150	6	32	4	1	*a*HNTs
14	150	6	32	4	2	*m*HNTs
15	150	6	32	4	3	*u*HNTs
16	150	8	28	6	1	*a*HNTs
17	150	8	28	6	2	*m*HNTs
18	150	8	28	6	3	*u*HNTs
19	160	4	32	6	1	*m*HNTs
20	160	4	32	6	2	*u*HNTs
21	160	4	32	6	3	*a*HNTs
22	160	6	28	8	1	*m*HNTs
23	160	6	28	8	2	*u*HNTs
24	160	6	28	8	3	*a*HNTs
25	160	8	30	4	1	*m*HNTs
26	160	8	30	4	2	*u*HNTs
27	160	8	30	4	3	*a*HNTs

## Appendix B

**Table B1:** Summary of the mechanical and physical property results.

No. Exp.	Tensile Strength (MPa)		Young’s Modulus (MPa)		Tensile Strain (%)		Hardness Shore A		Density (g/cm^3^)	
	Average	S/N	Average	S/N	Average	S/N	Average	S/N	Average	S/N
TPU	17.7	----	2.3	----	430.3	----	55.45	----	1.112	----
1	18.9	25.5	7.6	17.6	448.1	53.0	83.1	38.4	1.114	0.94
2	22.5	27.0	11.6	21.3	755.8	57.6	88.9	39.0	1.118	0.97
3	23.2	27.3	18.3	25.3	651.8	56.3	85.7	38.7	1.124	1.02
4	19.2	25.7	6.8	16.7	464.9	53.3	79.1	37.9	1.113	0.930
5	23.1	27.2	13.3	22.5	774.4	57.8	88.4	38.9	1.116	0.94
6	23.9	27.2	16.7	24.5	665.1	56.5	93.5	39.4	1.123	1.010
7	19.5	25.8	8.2	18.3	489.3	53.8	82.7	38.4	1.115	0.95
8	22.9	27.5	12.7	22.1	784.9	57.8	89.5	39.1	1.119	0.980
9	22.9	27.2	19.4	25.8	671.3	56.5	89.7	39.1	1.125	1.03
10	20.1	26.1	9.7	19.7	530.1	54.5	88.4	38.9	1.116	0.95
11	26.3	28.4	13.6	22.7	793.9	57.9	94.7	39.1	1.119	0.98
12	18.2	25.2	12.8	22.1	431.1	52.7	66.9	36.5	1.122	1.00
13	20.5	26.2	8.4	18.5	534.0	54.6	81.1	38.2	1.114	0.94
14	24.9	27.9	15.8	23.9	794.2	57.9	98.5	39.9	1.117	0.96
15	18.1	25.1	13.3	22.5	467.5	53.4	70.4	36.9	1.121	0.99
16	20.9	26.4	10.5	20.4	541.9	54.7	81.6	38.2	1.117	0.96
17	25.9	28.3	14.9	23.5	819.6	58.2	100.4	40.0	1.120	0.98
18	17.8	24.9	12.1	21.7	453.6	53.1	64.4	36.2	1.123	1.01
19	21.4	26.6	11.8	21.4	584.8	55.3	92.3	39.3	1.117	0.96
20	20.3	26.2	9.6	19.6	701.5	56.9	88.8	39.0	1.116	0.95
21	19.0	25.6	13.6	22.7	588.7	55.4	71.3	37.1	1.123	1.01
22	21.9	26.8	10.4	20.4	588.3	55.4	89.0	39.0	1.116	0.95
23	21.2	26.5	10.8	20.7	716.3	57.1	90.7	39.2	1.115	0.94
24	19.7	25.9	14.8	23.4	615.3	55.8	74.4	37.4	1.122	1.00
25	22.7	27.1	10.9	20.8	599.4	55.6	91.1	39.2	1.118	0.97
26	22.1	26.9	11.6	21.3	720.8	57.2	90.8	39.2	1.118	0.97
27	19.4	25.8	15.2	23.6	625.2	55.9	73.5	37.3	1.124	1.02

## Appendix C

**Table C1:** Response table for S/N ratios “larger is better” for responses.

	Parameter	Injection Temp. (°C)	Injection Pressure (bar)	Mold Temp. (°C)	Injection Time (min)	HNTs Loading (wt %)	HNTs Type
Tensile strength (MPa)	Level I	26.73	26.43	26.53	26.55	26.25	25.76
	Level II	26.52	26.52	26.62	26.46	27.33	26.42
	Level III	26.38	26.67	26.47	26.60	26.04	27.44
	Delta Δ	0.35	0.23	0.16	0.14	1.30	1.67
	Rank	3	4	5	6	2	1
Young’s modulus (MPa)	Level I	21.56	21.38	21.58	21.65	19.31	20.05
	Level II	21.67	21.45	21.54	21.43	21.96	21.59
	Level III	21.54	21.93	21.65	21.69	23.50	23.13
	Delta Δ	0.13	0.55	0.10	0.26	4.19	3.08
	Rank	5	3	6	4	1	2
Tensile strain (%)	Level I	55.85	55.52	55.69	55.72	54.46	54.51
	Level II	55.24	55.76	55.71	55.70	57.63	56.01
	Level III	56.06	55.88	55.76	55.74	55.07	56.65
	Delta Δ	0.82	0.36	0.07	0.04	3.17	2.14
	Rank	3	4	5	6	1	2
Hardness Shore A	Level I	38.76	38.48	38.45	38.52	38.61	37.96
	Level II	38.27	38.54	38.55	38.45	39.30	38.23
	Level III	38.51	38.51	38.53	38.55	37.62	39.34
	Delta Δ	0.49	0.06	0.10	0.10	1.67	1.38
	Rank	3	6	4	5	1	2
Density (g/cm^3^)	Level I	0.9731	0.9748	0.9748	0.9740	0.9498	0.9645
	Level II	0.9748	0.9645	0.9748	0.9740	0.9654	0.9748
	Level III	0.9748	0.9835	0.9731	0.9748	1.0076	0.9835
	Delta Δ	0.0018	0.0190	0.0017	0.0009	0.0578	0.0190
	Rank	4	3	5	6	1	2
